# Converting relative amplicon abundances to absolute abundances via flow cytometry: metagenomic validation and application to long ocean transects

**DOI:** 10.1093/ismeco/ycag081

**Published:** 2026-03-27

**Authors:** Nathan L R Williams, Qicheng Bei, Yubin Raut, Jed A Fuhrman

**Affiliations:** Department of Biological Sciences–Marine and Environmental Biology, University of Southern California, Los Angeles, CA 90026, United States; Department of Biological Sciences–Marine and Environmental Biology, University of Southern California, Los Angeles, CA 90026, United States; Department of Earth, Atmospheric, and Planetary Sciences, Massachusetts Institute of Technology, Cambridge, MA 02139, United States; Department of Biological Sciences–Marine and Environmental Biology, University of Southern California, Los Angeles, CA 90026, United States

**Keywords:** absolute microbial abundance, flow cytometry; amplicon sequencing correction, internal standard normalization, rRNA gene copy estimation, *Prochlorococcus* and *Synechococcus*, Atlantic Meridional transect (AMT), Global rRNA Universal Metabarcoding of plankton (GRUMP), *recA* and *radA* metagenomics, marine microbial ecology

## Abstract

With microbes critical for ocean ecological and biogeochemical processes, we need to understand their abundance and diversity distributions. While traditional amplicon sequencing provides only relative abundance data, and the strongly preferred absolute abundances can be determined from samples spiked with internal standards, few oceanographic studies with absolute abundances exist. However, many have flow cytometry (FCM) data that should allow us to retrospectively “anchor” the relative abundances into absolute abundances. We tested this hypothesis with data from the 29th Atlantic Meridional Transect (AMT29) cruise where we had FCM of *Synechococcus and Prochlorococcus*, amplicons corrected with internal standards, and absolute cell count estimates from single copy *recA* and *radA* metagenomics. Anchoring the AMT29 amplicon data with *Synechococcus* FCM (used because phycoerythrin in *Synechococcus* is reliably detected by FCM in surface waters) yielded results strongly correlated with amplicon data corrected with internal standards (Pearson’s *r* = 0.94, slope = 0.73), FCM (*r* = 0.80, slope = 0.43), and *recA*-based genome counts (Pearson’s *r* = 0.94, slope = 0.62). Seeing this method worked reasonably well, we then generated estimates of absolute rRNA gene abundances from the Global rRNA Universal Metabarcoding of Plankton (GRUMP) transects that had FCM data (Pacific ~65 N to ~40S). These FCM-anchored gene copy estimates also showed strong correlations to FCM data (i.e. anchor with *Synechococcus* and predict *Prochlorococcus*), with *r* values ranging from 0.48–0.86. While the results are clearly only reasonable estimates, we believe the approach has the potential to significantly enhance the value of amplicon data which have accompanying FCM data.

## Introduction

Sequencing of 16S and 18S amplicons to study the ecology of microbes in the environment yields taxonomically detailed data that is essential to the study of fundamental microbial ecological questions relating to diversity [1, 2], where taxa are and why, as well as beyond and into questions surrounding global biogeochemical cycles [3]. The overwhelming majority of marine microbial studies with this approach to date have been limited to reporting relative abundances, even though absolute abundances would be much more useful for studies of community ecology, ecosystems, and biogeochemical cycles. Changes in the relative abundance of a given taxon are dependent not only on that taxon but also on all the other taxa, leading to potential misleading patterns that cannot be fully corrected mathematically [4]. However, microbiome studies in various habitats have reported absolute abundances for several years, using quantitative PCR (qPCR) to anchor Dimethylsulfoniopropionate (DMSP) gene counts as proxies of genome counts of *Roseobacter* to then anchor 16S data [5], or in other studies, *HSP60* gene counts from qPCR as proxies for genome counts of *Vibrio* to anchor *HSP60* sequencing data [6] while other methods have included spike-in bacteria in soil studies [7, 8]. Even though internal standards have been used for many years in metatranscriptomic work [9], microbiome studies have applied internal DNA standards since 2016 [8, 10, 11], but only a few marine studies have used whole genome DNA standard “spike-ins,” starting with Lin *et al.* and Gifford *et al.* [12, 13]. In this approach, a known quantity of internal DNA standard, added prior to DNA extraction, is used to calculate a recovery ratio, enabling the calculation of estimated gene copies per volume for all amplicon sequence variants (ASVs) within the sample. This has since been applied in various studies [12–16] to quantify marine microbial community composition, and its accuracy has been verified by comparisons with *Prochlorococcus* FCM in Eastern Pacific Ocean samples, which reported a Pearson’s *r* of 0.99 and a slope of 1.01 in the scatterplot of absolute FCM counts vs calculated ASV abundance [14]. In addition, internal standards have been used with metagenomic data to estimate the absolute number of single-copy genes, extrapolated to haploid genome equivalents, which is an estimate of cell counts because most bacteria are thought to be haploid [13, 17].

Though clearly a major advance, the addition of DNA internal standards in marine microbial ecology is only now gaining traction, meaning that almost all previously published ocean data sets, including from the very recently published Global rRNA Universal Metabarcoding of Plankton (GRUMP) database [18], did not have internal standards added during DNA extraction. Therefore, we propose an alternative approach to produce estimated copies of ASVs per volume of seawater from prior datasets, by taking advantage of the fact that many oceanographic cruises with amplicon data also have contemporaneous data from FCM to measure abundances of *Prochlorococcus* and *Synechococcus.* Because these taxa are readily identifiable in ASVs, we can use the aggregated rRNA ASV count of either one (e.g. sum of all *Synechococcus* ASVs in a sample), along with the FCM data (cells per liter), to convert all relative ASV abundances into absolute ASV copy number estimates, via a “recovery ratio,” the ratio of observed cells per liter to observed number of ASVs in a sample. This ratio can be calculated for *Prochlorococcus* and/or *Synechococcus*, and applied to all other taxa, under the assumption that the amplicons are proportionally representative of organism abundance, supported (for the 515F-Y/926R primers) by published results from prokaryotic and eukaryotic mock communities [19, 20].

In this study, we wished to test the hypothesis that ASV data could be anchored with FCM data, with a primary aim of testing how robust this was compared to three other datasets from the same AMT29 cruise. We calculated recovery ratios with *Synechococcus* FCM along the 29th Atlantic Meridional Transect (AMT29) and compared the abundance from multiple prokaryotic organisms within this corrected data to the same organism absolute abundance obtained by three different methods (within the same samples): (i) FCM, (ii) amplicons with internal standards, as described in Jones-Kellett *et al.* [14], and (iii) genome equivalents calculated by *recA* for bacteria and *radA* for archaea from metagenomes, as described in Bei *et al.* [17]. We then applied this same approach to correct existing data from the GRUMP database by McNichol *et al.* [18] using *Synechococcus* FCM [because *Prochlorococcus* flow cytometry (FCM) can be unreliable in surface waters due to faint chlorophyll fluorescence] and compared the corrected *Prochlorococcus* ASVs to direct estimates of *Prochlorococcus* by FCM, as a second testbed of the approach.

## Materials and methods

### Sample collection

For the AMT29, water was collected at the surface between 2 and 5 m by Niskin bottle. For AMT29, 1 l of whole seawater was pumped through a 0.22 μm Sterivex filter, and then 500 μl RNA Later Solution (Invitrogen by Thermo Fisher Scientific) was added to the Sterivex filter for sample preservation prior to storage at −80°C. During the 2017 and 2019 (Simons Collaboration on Ocean Processes and Ecology) SCOPE-Gradients cruises (Gradients 2 and Gradients 3), 0.7–4 l of whole seawater was collected using the ship’s underway system, which is ~7 m below the surface. Filtered volumes for each sample were recorded. This water was filtered onto 0.22 μm, 25 mm Supor membrane filters and stored at −80°C until DNA extraction.

### DNA extraction

For AMT29, RNAlater® was centrifuged out of the Sterivex filter, and then the filter was rinsed with TE buffer. Suspended DNA was then desalted and recovered from RNAlater® and TE buffer mix by centrifugal ultrafiltration (Centricon, three cycles). Desalted nucleic acid was added back to the crude extract for further purification. Sterivex filter casings were cracked open using sterile (ethanol and flamed) pliers into petri dishes. The filter was removed from plastic housing, cut into small strips using sterile blades and forceps, and added to bead beating tubes along with the liquid nucleic acid fraction and RLT lysis buffer from the AllPrep DNA/RNA mini kit (Qiagen, Valencia, CA, USA). Cells were lysed using bead beating with equal amounts (0.15 g) of 0.1-, 0.2-, and 0.5-mm zircon beads, followed by total nucleic acid purification with the AllPrep DNA/RNA mini kit (Qiagen). For quantitative analysis, three genomic standards (*Thermus thermophilus* ATCC BAA-163, *Blautia producta* ATCC27340, and *Deinococcus radiodurans* ATCC13939) were added to the lysis buffer after bead beating (crude DNA extraction), targeting ~1% of total DNA content as internal standards. DNA extraction and purification generally followed manufacturer directions, with the detailed DNA extraction and purification protocol available at https://www.protocols.io/workspaces/fuhrman-lab. DNA from the SCOPE-Gradients cruises was isolated and purified following [21].

### Sequencing and amplicon production

Relative abundance information was generated by PCR amplification of extracted DNA using the 515Y (5′-GTGYCAGCMGCCGCGGTAA), 926R (5′-CCGYCAATTYMTTTRAGTTT) universal rRNA gene primer pair [20], which amplifies 16S, chloroplast 16S, and 18S rRNA simultaneously. All DNA samples were amplified using the protocol available at (https://www.protocols.io/view/fuhrman-lab-515f-926r-16s-and18s-rrna-gene-sequen-vb7e2rn). Mock communities were added to the sequencing run as described in Yeh *et al.* [20]. Briefly, PCR master mix consisted of 12 μl of PCR water (VWR), 10 μl of 5′ Master Mix (0.5 U Taq, 45 mM KCl, 2.5 mM Mg2+, 200 μm each dNTP), 1.5 μl of 1:1, 515 F:926 R barcoded primer mix (0.3 mM each primer), and 1 μl of DNA per reaction at a concentration of 1 ng/μl for a final volume of 25 μl. We used 5′ master mix (Quantabio) for SCOPE-Gradients 2 and SCOPE-Gradients 3 samples, and GoTaq master mix (Promega) for AMT29. PCR was performed under the following conditions: initial denaturing at 95°C for 120 seconds, followed by 30 cycles of 95°C for 45 seconds, 50°C for 45 seconds, and 68°C for 90 seconds, with a final elongation step at 68°C for 300 seconds. PCR products were stored at 4°C before being cleaned using the Agencourt AMPure XP PCR purification protocol. Finally, DNA concentration was quantified using the Pico-Green dsDNA Quant-iT Assay Kit. We used barcoded rRNA gene primers that had linkers so they made sequencing libraries directly. Pooled sequences for each run were cleaned and concentrated with SPRIselect (Beckman Coulter) beads. The cleaned pool was quantified using the Qubit dsDNA HS Assay Kit. Finally, the 16S and 18S rRNA amplicon concentrations in the pool were determined with a Bioanalyzer Chip: High Sensitivity DNA Kit.

For SCOPE-Gradients 2 and SCOPE-Gradients 3, sequencing was done using HiSeq Rapid Run technology (2 × 250 bp), while AMT29 was sequenced on the Element Biosciences AVITI at UMGC (2 × 300 bp). Sequences were demultiplexed using cutadapt (https://github.com/jcmcnch/demux-notes) and denoised to ASVs with a custom analysis pipeline based on QIIME2 and DADA2 [22, 23], which can be found on GitHub (https://github.com/jcmcnch/eASV-pipeline-for-515Y-926R), which uses a default MaxEE value (sequences with errors higher than this will be discarded) of 2 for both forward and reverse reads. We classified sequences with a naive bayes classifier using the QIIME2 plugin classify-sklearn with databases subset to the amplicon region (database construction steps available at https://github.com/jcmcnch/515F-Y_926R_database_construction). The main difference between this pipeline and standard workflows is that it contains an initial 16S/18S rRNA splitting step, which is accomplished using bbsplit against curated 16S and 18S rRNA databases derived from SILVA version 138.1 and PR2 version 4.14.0. This results in two subsets of data (16S and 18S rRNA) that are then denoised separately and later merged. Note that standard pipelines require a merging of forward and reverse reads where they overlap, which for these rRNA gene primers would lead to the removal of all 18S rRNA sequences because their forward and reverse 18S rRNA reads do not overlap. While the standard method (with overlap merging) was applied to the 16S sequences, the 18S rRNA sequences were concatenated. We used a universal trim length of 220 bp for forward and 180 bp for reverse reads before concatenation so that ASVs could be directly compared to each other across cruises. After denoising, sequencing depth was calculated, which ranged from 1–1 250 359, and we filtered any samples with a sequencing depth below 5000 out of the dataset, as they were considered too small to be statistically useful. On average, there were 180 528 sequences per sample before implementing the two corrections described below.

A correction was made to adjust for random variations in sample quality using the DADA2 output statistics, which took the nonchimeric reads (reads that passed the DADA2 chimera filter) and inflated them to the initial input number of reads using the following equation:


$$ p=\frac{n}{i} $$

Where *p =* proportion of reads which passed DADA2 chimera filter, *n* = nonchimeric reads that are output by the DADA2 chimera filter, and *i* = Initial input number of reads, which were put through the DADA2 filter. This was then multiplied by the ASV reads per ASV per sample to get *c*, which represents DADA2 corrected copies of each ASV. Copies per liter of ASVs using internal standards were then calculated by the following two equations:


$$ (i)\ x=\frac{c}{s}\kern0.75em (ii)\ Copies/L=\frac{c}{x} $$

Where for (i) *x* = recovery ratio, *c* = ASV reads recovered via sequencing and corrected for losses during DADA2 chimera removal, and *s* = internal standard genomes per liter added to sample (Supplemental Tables 1 and 2). Internal standard copies per liter (*s*) added were calculated using the following equation:


$$ \mathrm{S}=\frac{total\ internal\ standard\ added\ (ng)\times 6.022\times{10}^{23}}{660\times internal\ standard\ length\ (bp)\times 1\ x\ {10}^9} $$

Similar to copies per liter of ASVs using internal standards, copies per liter of ASVs using FCM were calculated by the following two equations:


$$ (i)\ x=\frac{a/2}{f}\kern0.5em (ii)\ Copies/L=\frac{c}{x} $$

Where for (i) *x* = recovery ratio, *a* = ASV reads recovered via sequencing and corrected for losses during DADA2 chimera removal that were assigned to *Synechococcus* and then divided by 2 to account for *Synechococcus* having two copies of 16S [24], and *f* = *Synechococcus* cells by FCM/L. We used *Synechococcus* over *Prochlorococcus* FCM because phycoerythrin pigments within *Synechococcus* are more easily detected by FCM in surface waters, where photo-acclimated *Prochlorococcus* produce very small amounts of photosynthetic pigment as they are in extremely high-light conditions. And then for (ii) *c* = ASV reads recovered via sequencing and corrected for losses during DADA2 chimera removal.

### Metagenomic cell counts using *recA* and *radA*

DNA libraries were produced with the NEBNext® Ultra™ II FS DNA Library Prep Kit (New England Biolabs). Metagenomic libraries were sequenced on an Illumina NovaSeq platform at the Tufts University Core Facility (Boston, MA, USA) using 2 × 250 bp paired-end sequencing. Absolute gene abundances were estimated using the method described in Bei *et al.* [13, 17]. Briefly, raw reads were quality-trimmed using Trimmomatic v0.39 [25]. Paired-end reads were assembled using PEAR v0.9.6 [26]. Reads from the internal genomic standards were identified via BLASTn (e-value <0.001, %id >95%, alignment length 50%, bit score > 50), followed by BLASTx searches (e-value <0.001, %id >98%, bit score > 50). Bacterial *recA* and archaeal *radA* proteins were downloaded from the RefSeq protein database (2024.11), and metagenome reads were compared to the databases using DIAMOND v2.1.9 [27] (BLASTx, −e 0.001, −k 1, %id >80%, bit score > 50). The cutoff of 80% identity to reference sequences was chosen because we did not want to limit results only to the reference sequences, but at the same time avoid counting related nontarget genes. Protein sequences were also verified with GhostKOALA against the Kyoto Encyclopedia of Genes and Genomes web server [28]. The taxonomies of *recA* and *radA* genes were determined by aligning the sequences to the NCBI nr database (2024.11) using DIAMOND (BLASTx, −e 1e-5, −k 20), and the outputs were summarized using MEGAN v7 community edition [29] with the GTDB taxonomy. For more details, see Bei *et al.* [17].

### Cell counts using flow cytometry

For AMT29 [30], 250 ml seawater samples were collected from Niskin bottles, stored at 4°C, and then analyzed within 2 hours of collection. Samples were measured using a Becton Dickinson FACSort flow cytometer to enumerate *Prochlorococcus* and *Synechococcus* cells/volume seawater. For SCOPE-Gradients 2 [31] and 3 [32], discrete samples for the abundance of cyanobacteria were collected by CTD-rosette and measured with an Influx flow cytometer as described in Leifer *et al.* [33].

### Statistical analyses and data visualization

All statistical analyses and visualizations were performed in R v4.3.245 [34] using tidyverse [35], ggpubr [36], stringr [37], Cairo [38], and patchwork [39] packages. All packages were used with their default settings. Scripts used to arrange data, perform statistics, and create figures can be found at (https://github.com/Nwilliams96/Project-5-Anchoring-ASVs-with-Flow-Cytometry).

## Results and discussion

**Figure 1:**
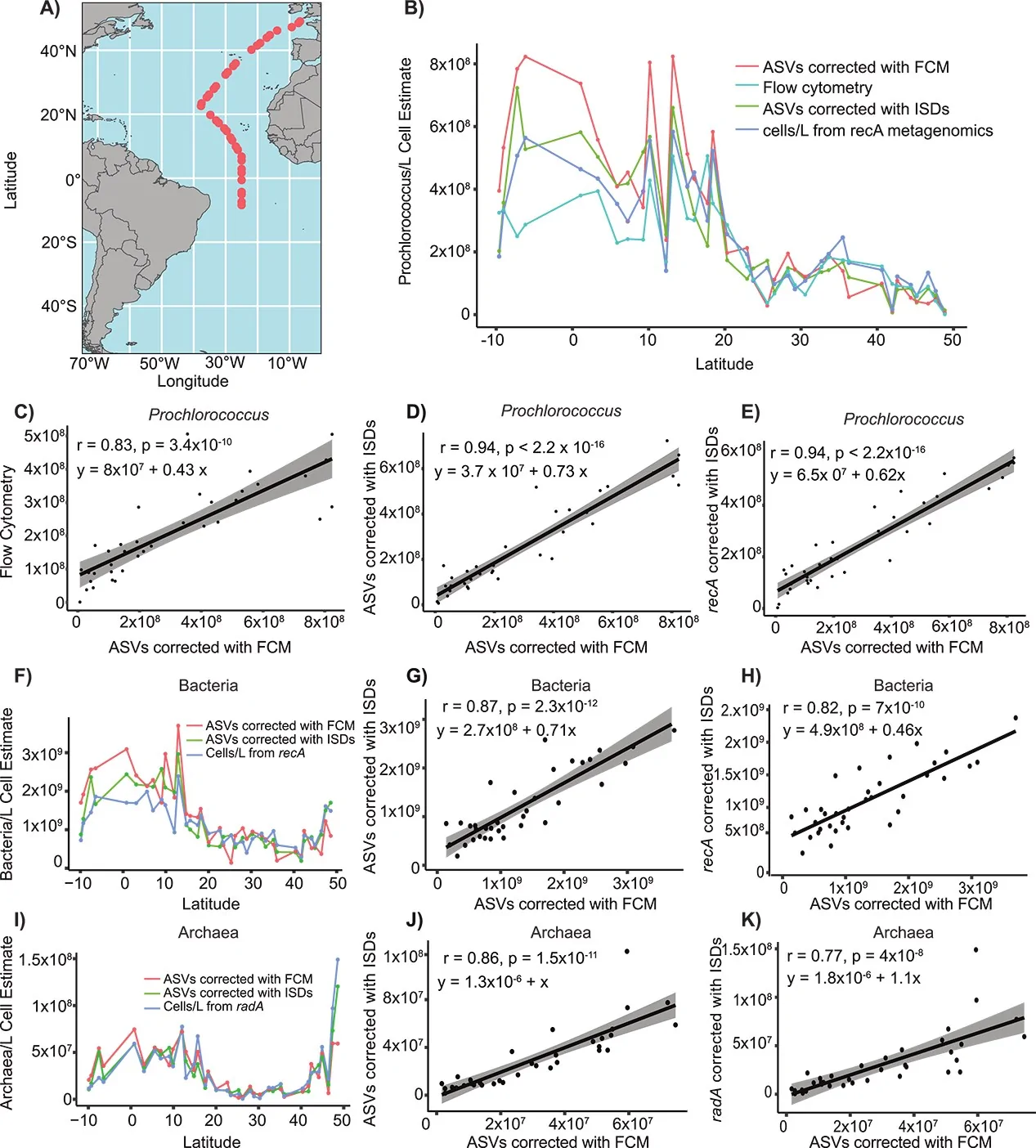
(A) Map of AMT samples taken. (B) Line plot of *Prochlorococcus* cell and gene copy estimates by (red) ASVs corrected with *Synechococcus*-FCM, (blue) FCM, (green) ASVs corrected with internal standards, and (purple) genome copy estimates from *recA* genes that were assigned to *Prochlorococcus* through metagenomic analysis. (C–E) regressions of *Prochlorococcus* abundances derived from *Synechococcus*-FCM corrected amplicons and abundances derived from (C) FCM, (D) internal standard corrected amplicons, and (E) metagenomically derived *recA* genome equivalents. Each plot has the Pearson’s correlation reported (top) and equation of the regression reported (below). (F) Line plot of bacteria abundance estimates per liter of seawater across latitude by (red) ASVs corrected with FCM, (green) ASVs corrected with internal standards, and (purple) genome copy estimates from *recA* genes that were assigned to *Prochlorococcus* through metagenomic analysis. (G) Linear regressions of total bacteria abundance from ASV copies/L corrected with internal standards against ASV copies/L corrected with FCM. (H) Linear regressions of total bacteria abundance from *recA* metagenomics displaying cells/L corrected with internal standards against ASV copies/L corrected with FCM. (I) Line plot of archaea abundance estimates per liter of seawater across latitude by (red) ASVs corrected with FCM, (green) ASVs corrected with internal standards, and (purple) genome copy estimates from *recA* genes that were assigned to *Prochlorococcus* through metagenomic analysis. (J) Linear regressions of total archaea abundance from ASV copies/L corrected with internal standards against ASV copies/L corrected with FCM. (K) Linear regressions of total archaea abundance from *radA* metagenomics displaying cells/L corrected with internal standards against ASV copies/L corrected with FCM.

The primary objective of this work was to assess the quality of absolute rRNA gene abundance estimates made via anchoring the relative ASV abundances with flow cytometric data, by applying such anchoring to ocean transect data and comparing to independent absolute abundance estimates. To make the FCM-anchored estimates for microbial taxa along the AMT29 cruise, we multiplied the amplicon abundances by the *Synechococcus* FCM recovery ratio to yield gene copies per liter (described in methods). And because we have multiple other ways of estimating absolute abundances from the AMT-29 samples, we were able to make several kinds of comparisons. For the first approach, we took advantage of having FCM data also available for *Prochlorococcus*, so we compared *Synechococcus*-corrected ASV abundances of *Prochlorococcus* to *Prochlorococcus* direct FCM estimates. We did so by selecting ASVs assigned to *Prochlorococcus*, summing their abundance and then comparing this abundance by sample to *Prochlorococcus* ASVs per liter. For the second approach, we compared the *Synechococcus*-FCM-corrected abundances to rRNA amplicon abundances corrected with genomic internal standards, a method which had been previously shown to be highly accurate for marine cyanobacteria on a Pacific transect [14]. And for the third approach, we compared the ASV abundances corrected by *Synechococcus* FCM to haploid genome equivalent abundances estimated by single-copy *recA* gene abundances corrected with genomic internal standards, as recently reported for this same transect [17] (Fig. 1B–E). The ASV abundances corrected using FCM and ASV abundances corrected with internal standards showed the strongest relationship among all comparisons, with a Pearson’s r of 0.94 and a slope of 0.73 (Fig. 1D). *Prochlorococcus* ASVs corrected with *Synechococcus* FCM also had strong correlations with the *Prochlorococcus* estimated via *recA* (Pearson’s *r* = 0.94, slope = 0.62), and with *Prochlorococcus* estimated directly by FCM (Pearson’s *r* = 0.83, slope = 0.43) (Fig. 1C and E). However, we noted that the slopes of these regressions, which would be 1 with a perfect relationship, were less than 1, indicating underestimates of the *y* values in these regressions compared to x values.

There are a few possible, nonmutually exclusive, explanations for such underestimates, and we will discuss the ones we think are most likely. One potential explanation is that the FCM was under-estimating *Prochlorococcus* in surface waters where these samples were collected. This phenomenon has been noted previously, following the observation that near-surface *Prochlorococcus* cells produce very small amounts of photosynthetic pigment as they are photo-acclimated in extremely high-light conditions, resulting in their autofluorescence being too faint for reliable detection by traditional FCM [40]. And detectability relates to the particular FCM instrument used, with some more sensitive than others; hence one may expect differences between cruises where different flow cytometers were used. Dim fluorescence has not been reported as a problem for *Synechococcus*, however, because this genus has the accessory pigment phycoerythrin that is more easily detected, and this is a reason we use this genus for anchoring our absolute abundance estimates. Note that historically, it is thought that the dim fluorescence of *Prochlorococcus* in the surface layers contributed to their escaping detection by traditional epifluorescence microscopy and early FCM onboard measurements [40]. More recently, this underestimation has been discussed by others, including Hartmann *et al.* [41] who used a BD FACSort flow cytometer on an earlier AMT cruise, AMT4 (the same model was used for AMT29 in this study). A previous investigation conducted in overlapping regions of the South Atlantic Ocean reported that a compact, shipboard instrument with fewer photomultipliers (BD Accuri™ C6) severely underestimated *Prochlorococcus* abundances relative to a high-sensitivity, laboratory-based instrument (BD FACSCanto™), while producing comparable data for heterotrophic bacteria and eukaryotic phytoplankton [42].

A second possible explanation for possible underestimates of particular taxa in this study, notably those from internal-standard corrected *recA* and *radA* counts, involves the difficulty of comprehensively characterizing and identifying metagenomic reads originating from *recA* and *radA.* While there is a good database for these taxa, it still does not cover the full breadth of natural marine microbial diversity, and it would not be surprising if many *recA* or *radA* reads miss being annotated as such. Note for this approach we used an 80% nucleotide identity cutoff to call these gene identities, chosen to avoid erroneously calling reads from related genes, but potentially missing true ones. We expect gene databases to improve over time, allowing better classification in the future. Note also that Bei *et al.* [17] showed that with data from AMT29, FCM and *recA* total cyanobacteria had a correlation and slope much higher than what we observed between ASV and cell count data (Pearson’s *r* = 0.99; slope = 1.03), suggesting the likelihood that both *recA* and FCM underestimated *Prochlorococcus* to similar extents on this cruise.

A third issue in making comparisons is that gene abundances are affected by gene copies per genome. It is well known that rRNA genes can occur in multiple copies [43, 44], especially for copiotrophic taxa, and this needs to be considered when comparing to *recA* and *radA* abundances interpreted as haploid genomes per liter (due to these being “single-copy genes”), as well as FCM counts. In our case, ASV copies per liter would be expected to be higher than cell counts derived by FCM and genome equivalents derived by *recA* to the extent that some *Prochlorococcus* within the transect (such as *P. marinus MIT 9303* [45]) may have more than one copy of the 16S gene per cell. This could help explain why the *recA* derived genome equivalents per liter and FCM cells per liter were consistently lower than the ASV copies per liter, which was observed along most of the AMT29 transect (Fig. 1B).

A fourth possible explanation, which would be relevant only for the few cases that the molecular methods yielded lower estimates than FCM, could be that the DNA extraction biased against some cyanobacteria, e.g. by not fully lysing certain cells [46]. We aimed to minimize the potential for such biases by maximizing lysis through bead beating for 5-minutes with 0.1-, 0.2-, and 0.5-mm zircon beads to create a spectrum of impact energy and contact geometries, maximizing the disruption of diverse cell walls and membranes. While we recognize that DNA extractions have biases between methods [47], it has been shown that methods which employee bead beating obtain a higher DNA yield and more ASVs than ones which employee chemical lysis only [47].

Finally, it should also be noted that a potential reason for the amplicon data absolute estimates being higher than the FCM estimates of *Prochlorococcus* is that the primer pair over-amplified *Prochlorococcus* compared to other taxa. Even though the mock community samples included in our PCR and sequencing analyses has a member of *Prochlorococcus* accurately reflected quantitatively in sequences (Supplementary Fig. 5), this is only one representative *Prochlorococcus* of many. Primers are known to both over and under amplify their target genes [48], and so it is possible that some *Prochlorococcus* are over amplified in comparison to others, causing inflated results.

We observed similar trends relating to underestimations when comparing total bacteria ASVs corrected by FCM and ASVs corrected by internal standards (Pearson’s *r* = 0.87; slope = 0.71) (Fig. 1G), which had a slope much closer to 1 than the ASVs corrected with FCM and the *recA* data (Pearson’s *r* = 0.82; slope = 0.46) (Fig. 1H). Furthermore, the slopes also showed marked deviations from a 1:1 relationship when comparing ASVs corrected with FCM and *recA* among *Rhodobacterales, Flavobacteriales, Actinomarinales, SAR11,* and *Puniceispirillales* (0.33–0.75 for *recA* compared to 0.66–0.84 for ASVs corrected by internal standards) (Supplementary Figs. 1 and 2A–I). While this may again be in large part a copy number issue, assessing 16S rRNA operon copy numbers in these heterotrophic lineages is challenging, as comprehensive data remain limited—particularly for less well-characterized groups such as *Actinomarinales* and *Puniceispirillales*. However, copy number variation does not explain why there is a slope of 0.54 when comparing SAR11 FCM corrected ASVs to SAR11 from *recA* because SAR11 is widely known for its streamlined genome characterized by low numbers of paralogs, resulting in minimal gene redundancy and few duplicated genes, e.g. 16S rRNA ~1 [49]. Given that we assessed our amplicons with a mock community that included SAR11, which had minimal PCR bias (Supplementary Fig. 5), this suggests that much of the problem relates to the lack of a comprehensive *recA* database (hence, some *recA* reads are not annotated as such), and possibly also calling for alternative taxonomy assignment strategies, such as phylogenetic read placement of metagenomic reads onto phylogenetic trees that include contigs assembled from the environment [49].

By far, the best relationship we observed was among the total archaea, with comparisons strong between both the ASVs corrected with internal standards (Pearson’s *r* = 0.86; slope = 1) and the *radA* data (Pearson’s *r* = 0.77; slope = 1.1) (Fig. 1I–K). A similar result was also observed within a subset of the marine archaea, *MGII*, also when comparing to both ASVs corrected with internal standards (Pearson’s *r* = 0.78; slope = 1.1) and the *radA* data (Pearson’s *r* = 0.87; slope = 1) (Supplementary Fig. 3). The slopes were much better than what was observed within the bacteria data, probably because archaea generally have a low copy number of 16S (~1 per cell) [50] and have fewer and less divergent marine plankton lineages, making them easier to assign to *radA*, than bacteria to *recA*.

**Figure 2:**
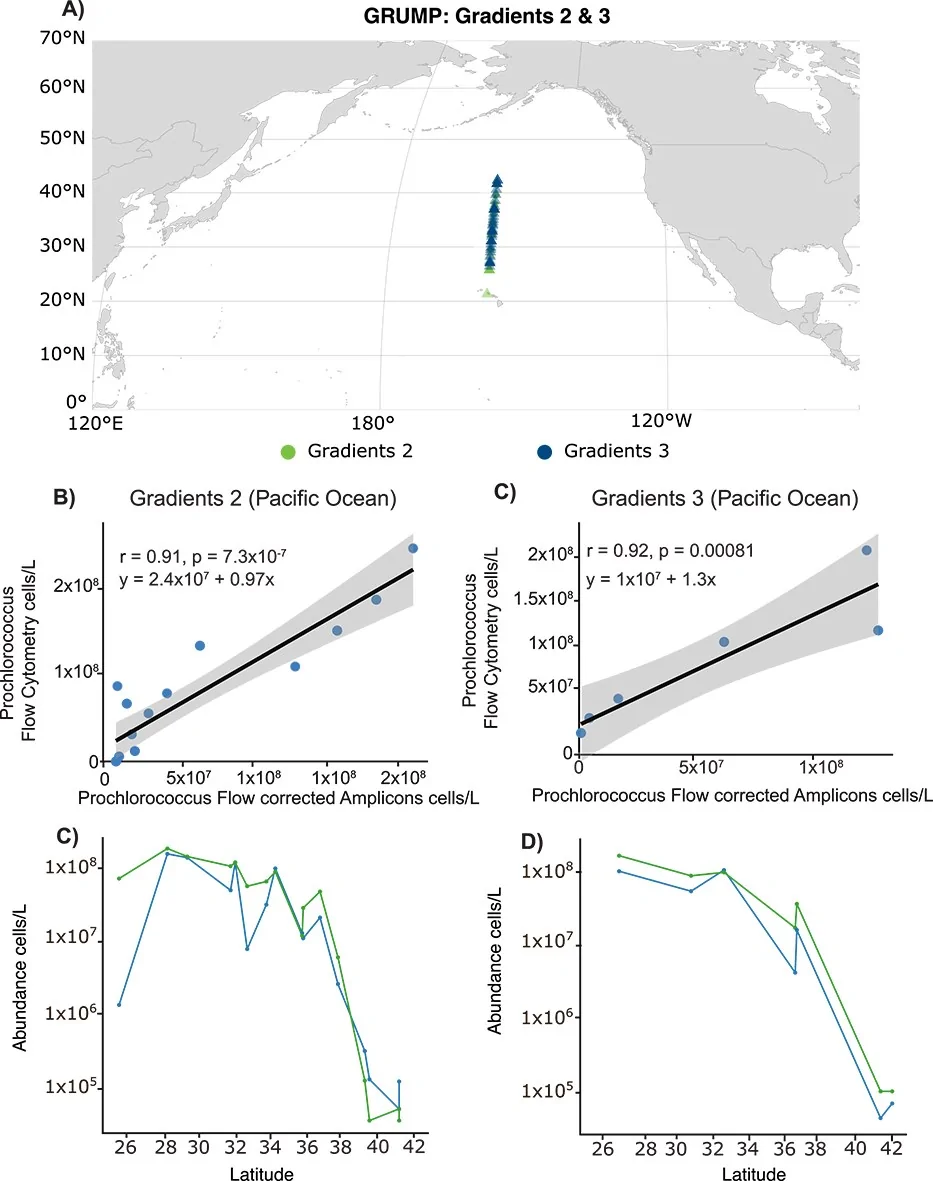
(A) Map of the Gradients 2 and 3 transects from GRUMP, i.e. the ones for which we used FCM in this study to yield absolute estimates. (B and C) *Prochlorococcus* cells/L measured by FCM compared to *Prochlorococcus* ASVs copies/L corrected with *Synechococcus* FCM, from original SCOPE-Gradients GRUMP transects. (C and D) Line plots of *Prochlorococcus* FCM (green) and total counts of copies of *Prochlorococcus* ASVs per liter of seawater, corrected with *Synechococcus* FCM for cruises (C) Gradients 2, (D) Gradients 3.

Since the AMT29 data showed strong correlations among methods, we proceeded to extend the FCM-corrected absolute ASV abundance estimates to samples from the recently published GRUMP dataset by McNichol *et al.* [18]. We downloaded FCM data from two GRUMP cruises that had collected influx FCM data, specifically SCOPE-Gradients 2 and 3, which are available on Simons CMAP [51] (Fig. 2A). For the SCOPE-Gradients 2 and 3 GRUMP transects, we found that correcting the data with *Synechococcus* yielded consistent trends between *Prochlorococcus* from flow corrected amplicons and *Prochlorococcus* FCM, as represented by high *r* values (Fig. 2A and B) (SCOPE-Gradients 2–Pearson’s *r* = 0.84, slope = 0.97, SCOPE-Gradients 3 – Pearson’s *r* = 0.86, slope = 1.3). These data were comparable to the Pearson’s r values and slopes that were observed in Jones-Kellett *et al.* [14] who reported a slope of 1.01 and a Pearson’s r of 0.9942 when comparing *Prochlorococcus* ASVs corrected by internal standards and *Prochlorococcus* FCM taken during SCOPE-Gradients 4. This slope was much closer to 1 than what we reported above when comparing the *Prochlorococcus* ASVs corrected with FCM to *Prochlorococcus* FCM from AMT29 (slope = 0.43, Fig. 1C). In fact, the same comparison from AMT29, was less consistent between methods also (Fig. 1C) shown by lower Pearson’s *r* values and slopes in comparison to the SCOPE-Gradients cruises (AMT29—Pearson’s *r* = 0.83, slope = 0.43). These comparisons should be considered in the context of possible causes of underestimations noted earlier, notably the difficulty counting *Prochlorococcus* in oligotrophic near-surface waters. Optimizing their counts requires either higher laser intensities [52] or a flow cytometer such as an Influx, which notably was used by both SCOPE-Gradients cruises, that are equipped with unique forward scatter detectors that improve the forward scatter photomultiplier tube sensitivity to smaller particles [53]. The high correlations and slopes closer to 1 in the SCOPE-Gradients cruises (compared to AMT29) are consistent with the conclusion that the counts from the Influx flow cytometer are more accurate than that on the AMT29 cruise and more suitable for these ASV corrections. This highlights the critical importance of considering instrument sensitivity, precision, and detection limits (in addition to choosing *Synechococcus* as anchor) when leveraging FCM data to estimate absolute ASV abundances from relative amplicon sequences, and in interpreting the results.

Once the GRUMP data were corrected, we then subset ASVs from other taxa such as copiotrophic and oligotrophic bacterial species, as well as MGII Archaea (Supplementary Fig. 4). If the primary limitation is indeed the underestimation of *Prochlorococcus* by the FACSort FCM (the flow cytometer used to measure FCM for AMT29), then the values shown in Supplementary Fig. 4 can still be interpreted as estimates of ASV copies per volume of seawater. We acknowledge that the eukaryote data (Supplementary Fig. 4), while absolute 18S gene copies, still bear the issue of many eukaryotes, such as dinoflagellates, diatoms, copepods, and ciliates having 18S copy numbers that can range orders of magnitude [54, 55]. This means that while absolute 18S copy numbers, the data are very hard to interpret in terms of numbers of actual organisms. To address this, future work may include creating a database of gene copy numbers by dividing absolute amplicon data by *psbO* counts (recently reported “single copy” gene for prokaryotic and eukaryotic phytoplankton [17]) derived from the same organisms from within the same samples. For taxa known to possess a single copy of the 16S rRNA gene, such as *SAR11*, these values may further be interpreted as estimates of cell abundance per volume of seawater. The upshot is that these data are estimates of absolute copy number per liter—directly related to absolute cell counts—which have much more relevance to microbial ecologists, allowing them to determine where the total abundances of microbes vary between samples [16], as well as other end users such as modelers [56, 57], biogeochemists, and statisticians who prefer to work in the realm of absolute numbers rather than relative abundances that currently dominate existing marine microbial diversity data.

## Conclusion

The use of cyanobacterial FCM to convert relative to absolute microbial abundances has its caveats, such as there being few *Prochlorococcus* or *Synechococcus* outside of the euphotic zone or in polar regions, which prevents extending this approach to many important parts of the global ocean. Also, many DNA studies use size fractionated samples, and *Synechococcus* in particular (typically ranging 0.8–1.5 μm in diameter) may straddle between size fractions, potentially invalidating its use. The most impactful caveat, however, is that there were moderate discrepancies (fairly consistent underestimates) between corrections made via FCM and by genomic internal standards recovery ratios, where we generated absolute estimates of Bacteria that were fairly consistently ~70% of the bacterial ASV abundance, though much better for Archaea (assuming ASVs corrected with internal standards are accurate, as reported for cyanobacteria by Jones-Kellett *et al.* [14]). We suggest that where possible, it is best practice to make use of genomic internal standards to obtain ASV copies per volume seawater. However, if ASV corrected with FCM data is used, care must be taken in interpreting the data and extending the use of the absolute estimates. Based on our analysis, we conclude that Influx flow cytometers provide more reliable cell counts of cyanobacteria (most notably *Prochlorococcus*), and thus, would be the preferred instrument type for this approach. However, we recognize that most oceanographic cruises have employed other flow cytometer models less likely to accurately measure *Prochlorococcus*. Beyond using *Synechococcus* as we have done here, other alternative approaches may be called for, e.g. regression against other kinds of counts such as stained heterotrophic bacteria, as described in Cram *et al.* [58] and in any case the estimates must be evaluated with caution. Nonetheless, we argue that if used with care, our approach has the potential to significantly enhance the value and utility of historical amplicon datasets when they are paired with accompanying FCM data.

## Supplementary Material

Anchoring_Amplicons_with_Flow_Cytometry-Supp-R1_ycag081

## Data Availability

GRUMP data are available at Simons CMAP (https://simonscmap.com/catalog/datasets/GRUMP). The forward and reverse sequences were submitted to the NCBI database under accession numbers GA02/ GA10: PRJNA1194189, GA03: PRJNA1194192, GP13: PRJNA1195113, SCOPE-Gradients 2: PRJNA1195115, SCOPE -Gradients 3: PRJNA1196422, P16N: PRJNA1196483, P16S: PRJNA1196490, P15S: PRJNA1196498, HEOBI: PRJNA1196504, IND-2017: PRJNA1196513, K-AXIS: PRJNA119651, POTATOE: PRJNA1198072, I08S: PRJNA1198088, I09N: PRJNA1198607, MOSAiC: PRJNA1198992, and FRAM: PRJNA1199044. Raw sequence data of AMT29 have been deposited to the NCBI under accession: amplicon sequencing (PRJNA1226253) and metagenome with internal standards (PRJNA1194529 and PRJNA1194620). Databases of the single-copy genes (*radA* and recA) used for this study can be accessed through Figshare (https://doi.org/10.6084/m9.figshare.28921349.v1). The scripts used for metagenomics with internal genomic standards are available at https://github.com/beiqicheng. The scripts used in the analysis for this paper can be found at https://github.com/Nwilliams96.
