# Correction: Converting relative amplicon abundances to absolute abundances via flow cytometry: metagenomic validation and application to long ocean transects

**DOI:** 10.1093/ismeco/ycag274

**Published:** 2026-10-14

This is a correction to: Nathan L R Williams, Qicheng Bei, Yubin Raut, Jed A Fuhrman, Converting relative amplicon abundances to absolute abundances via flow cytometry: metagenomic validation and application to long ocean transects, *ISME Communications*, Volume 6, Issue 1, January 2026, ycag081, https://doi.org/10.1093/ismeco/ycag081

A change has been made to the originally published paper.

The following has been added to the **Acknowledgements** section: “We thank Enrico Ser-Giacomi, Jesse McNichol and Mick Follows for valuable early discussions of the conceptual basis and general formula for an FCM-based correction, including the terminology “anchor” and highlighting differences between Synechococcus and Prochlorococcus anchors.”

