# Supplementary material for: Converting relative amplicon abundances to absolute abundances via flow cytometry: metagenomic validation and application to long ocean transects
Source: ISME Commun. 2026 Mar 27;6(1):ycag081. doi: 10.1093/ismeco/ycag081 (PMC13157830; doi:10.1093/ismeco/ycag081)
Supplement: Anchoring_Amplicons_with_Flow_Cytometry-Supp-R1_ycag081 [file anchoring_amplicons_with_flow_cytometry-supp-r1_ycag081.docx]

**Converting Relative Amplicon Abundances to Absolute Abundances via Flow Cytometry: Metagenomic Validation and Application to Long Ocean Transects**

Nathan L R Williams^1*^, Qicheng Bei^1^, Yubin Raut^2^, Jed Fuhrman^1^

1. Department of Biological Sciences–Marine and Environmental Biology, University of Southern California, Los Angeles, California, United States

2. Department of Earth, Atmospheric, and Planetary Sciences, Massachusetts Institute of Technology, Cambridge, MA 02139, United States

*Corresponding Author

**Table 1.** Internal standards added to AMT29 samples which enabled calculation of recovery ratios.

|  | *Thermus thermophilus* HB27 | *Blautia producta* ATCC 27340 | *Deinococcus radiodurans* ATCC 13939 |
| --- | --- | --- | --- |
| Name | ATCC® BAA-163™ | ATCC® 27340™ | ATCC® 13939™ |
| Length | 2,143,708 nt | 6,200,054 nt | 3,279,485 nt |
| Number of CDS | 2244 | 5714 | 3148 |

**Table 2.** Internal standards added to each sample. All samples are surface samples taken from 5m.

| Sample ID | Latitude | Longitude | Depth | Internal standard ng | Each ng | *Thermus* copies/sample | *Blautia* copies/sample | *Deinococcus* copies/ sample |
| --- | --- | --- | --- | --- | --- | --- | --- | --- |
| DNA-tag-2 | 49.0 | -6.7 | 2 | 156 | 52 | 2.26E+07 | 7.91E+06 | 1.49E+07 |
| DNA-tag-3 | 48.5 | -7.2 | 5 | 53.1 | 17.7 | 7.71E+06 | 2.69E+06 | 5.06E+06 |
| DNA-tag-4 | 47.2 | -9.3 | 5 | 103.5 | 34.5 | 1.50E+07 | 5.25E+06 | 9.86E+06 |
| DNA-tag-5 | 46.2 | -13.9 | 5 | 3.03 | 1.01 | 4.40E+05 | 1.54E+05 | 2.89E+05 |
| DNA-tag-6 | 44.7 | -16.2 | 10 | 115 | 38.33 | 1.67E+07 | 5.83E+06 | 1.10E+07 |
| DNA-tag-7 | 43.9 | -17.1 | 5 | 97.8 | 32.6 | 1.42E+07 | 4.96E+06 | 9.32E+06 |
| DNA-tag-8 | 42.2 | -19.2 | 5 | 108.3 | 36.1 | 1.57E+07 | 5.49E+06 | 1.03E+07 |
| DNA-tag-9 | 41.5 | -20.2 | 2 | 102.9 | 34.3 | 1.49E+07 | 5.22E+06 | 9.81E+06 |
| DNA-tag-10 | 40.2 | -21.9 | 2 | 50.1 | 16.7 | 7.27E+06 | 2.54E+06 | 4.77E+06 |
| DNA-tag-11 | 35.9 | -26.9 | 5 | 1.53 | 0.51 | 2.22E+05 | 7.76E+04 | 1.46E+05 |
| DNA-tag-12 | 35.1 | -27.6 | 2 | 1.32 | 0.44 | 1.92E+05 | 6.70E+04 | 1.26E+05 |
| DNA-tag-13 | 33.2 | -29.3 | 5 | 99.6 | 33.2 | 1.45E+07 | 5.05E+06 | 9.49E+06 |
| DNA-tag-14 | 32.4 | -30.0 | 10 | 103.8 | 34.6 | 1.51E+07 | 5.27E+06 | 9.89E+06 |
| DNA-tag-15 | 28.8 | -33.0 | 5 | 59.1 | 19.7 | 8.58E+06 | 3.00E+06 | 5.63E+06 |
| DNA-tag-16 | 28.0 | -33.7 | 5 | 64.5 | 21.5 | 9.36E+06 | 3.27E+06 | 6.15E+06 |
| DNA-tag-17 | 26.1 | -35.2 | 5 | 28.8 | 9.6 | 4.18E+06 | 1.46E+06 | 2.74E+06 |
| DNA-tag-18 | 25.3 | -35.9 | 5 | 74.7 | 24.9 | 1.08E+07 | 3.79E+06 | 7.12E+06 |
| DNA-tag-19 | 23.4 | -37.5 | 5 | 103.2 | 34.4 | 1.50E+07 | 5.24E+06 | 9.83E+06 |
| DNA-tag-20 | 22.6 | -37.7 | 5 | 0.85 | 0.28 | 1.23E+05 | 4.31E+04 | 8.09E+04 |
| DNA-tag-21 | 19.8 | -34.8 | 5 | 2.36 | 0.79 | 3.42E+05 | 1.19E+05 | 2.24E+05 |
| DNA-tag-22 | 18.1 | -33.0 | 5 | 7.2 | 2.4 | 1.05E+06 | 3.65E+05 | 6.86E+05 |
| DNA-tag-23 | 17.3 | -32.2 | 5 | 16.05 | 5.35 | 2.33E+06 | 8.14E+05 | 1.53E+06 |
| DNA-tag-24 | 15.6 | -30.4 | 5 | 11.92 | 3.97 | 1.73E+06 | 6.05E+05 | 1.14E+06 |
| DNA-tag-25 | 14.7 | -29.7 | 5 | 51 | 17 | 7.40E+06 | 2.59E+06 | 4.86E+06 |
| DNA-tag-26 | 12.7 | -28.5 | 2 | 3.77 | 1.26 | 5.47E+05 | 1.91E+05 | 3.59E+05 |
| DNA-tag-27 | 11.8 | -28.0 | 5 | 3.54 | 1.18 | 5.14E+05 | 1.80E+05 | 3.37E+05 |
| DNA-tag-28 | 9.9 | -26.8 | 5 | 4.54 | 1.51 | 6.59E+05 | 2.30E+05 | 4.33E+05 |
| DNA-tag-29 | 8.9 | -26.2 | 2 | 3.44 | 1.15 | 4.99E+05 | 1.75E+05 | 3.28E+05 |
| DNA-tag-30 | 6.7 | -25.0 | 5 | 4.72 | 1.57 | 6.85E+05 | 2.39E+05 | 4.50E+05 |
| DNA-tag-31 | 5.5 | -25.0 | 5 | 5.52 | 1.84 | 8.01E+05 | 2.80E+05 | 5.26E+05 |
| DNA-tag-32 | 2.9 | -25.0 | 2 | 3.55 | 1.18 | 5.15E+05 | 1.80E+05 | 3.38E+05 |
| DNA-tag-33 | 1.7 | -25.0 | 2 | 3.84 | 1.28 | 5.57E+05 | 1.95E+05 | 3.66E+05 |
| DNA-tag-34 | -0.6 | -25.0 | 2 | 3.96 | 1.32 | 5.75E+05 | 2.01E+05 | 3.77E+05 |
| DNA-tag-36 | -4.3 | -25.0 | 2 | 0.25 | 0.08 | 3.63E+04 | 1.27E+04 | 2.38E+04 |
| DNA-tag-37 | -5.0 | -25.0 | 2 | 3.66 | 1.22 | 5.31E+05 | 1.86E+05 | 3.49E+05 |
| DNA-tag-38 | -7.4 | -25.0 | 2 | 3.71 | 1.24 | 5.39E+05 | 1.88E+05 | 3.54E+05 |
| DNA-tag-35 | -8.4 | -25.0 | 2 | 4.49 | 1.5 | 6.52E+05 | 2.28E+05 | 4.28E+05 |
| DNA-tag-39 | -10.7 | -25.0 | 2 | 3.06 | 1.02 | 4.44E+05 | 1.55E+05 | 2.92E+05 |
| DNA-tag-40 | -11.9 | -25.0 | 2 | 2.92 | 0.97 | 4.24E+05 | 1.48E+05 | 2.78E+05 |
| DNA-tag-41 | -14.3 | -25.0 | 2 | 0.05 | 5.1 | 2.22E+06 | 7.76E+05 | 1.46E+06 |
| DNA-tag-42 | -15.4 | -25.0 | 2 | 2.6 | 0.87 | 3.77E+05 | 1.32E+05 | 2.48E+05 |
| DNA-tag-43 | -20.2 | -25.0 | 2 | 2.19 | 0.73 | 3.18E+05 | 1.11E+05 | 2.09E+05 |
| DNA-tag-44 | -21.4 | -24.8 | 2 | 1.98 | 0.66 | 2.87E+05 | 1.00E+05 | 1.89E+05 |
| DNA-tag-45 | -23.7 | -24.9 | 2 | 4.45 | 1.48 | 6.46E+05 | 2.26E+05 | 4.24E+05 |
| DNA-tag-46 | -24.7 | -25.2 | 2 | 2.87 | 0.96 | 4.17E+05 | 1.46E+05 | 2.73E+05 |
| DNA-tag-47 | -26.8 | -25.8 | 2 | 3.27 | 1.09 | 4.75E+05 | 1.66E+05 | 3.12E+05 |
| DNA-tag-48 | -27.7 | -26.1 | 2 | 2.29 | 0.76 | 3.32E+05 | 1.16E+05 | 2.18E+05 |
| DNA-tag-49 | -30.0 | -26.8 | 2 | 3.63 | 1.21 | 5.27E+05 | 1.84E+05 | 3.46E+05 |
| DNA-tag-50 | -31.2 | -27.2 | 2 | 1.33 | 0.44 | 1.93E+05 | 6.75E+04 | 1.27E+05 |
| DNA-tag-51 | -33.6 | -27.9 | 2 | 3.48 | 1.16 | 5.05E+05 | 1.77E+05 | 3.32E+05 |
| DNA-tag-52 | -34.8 | -28.3 | 2 | 2.47 | 0.82 | 3.59E+05 | 1.25E+05 | 2.35E+05 |
| DNA-tag-53 | -37.1 | -29.0 | 2 | 6.14 | 2.05 | 8.91E+05 | 3.11E+05 | 5.85E+05 |
| DNA-tag-54 | -40.4 | -31.0 | 3 | 4.7 | 1.57 | 6.82E+05 | 2.38E+05 | 4.48E+05 |
| DNA-tag-55 | -41.9 | -35.4 | 2 | 4.71 | 1.57 | 6.84E+05 | 2.39E+05 | 4.49E+05 |

**2.0 – Results**

**
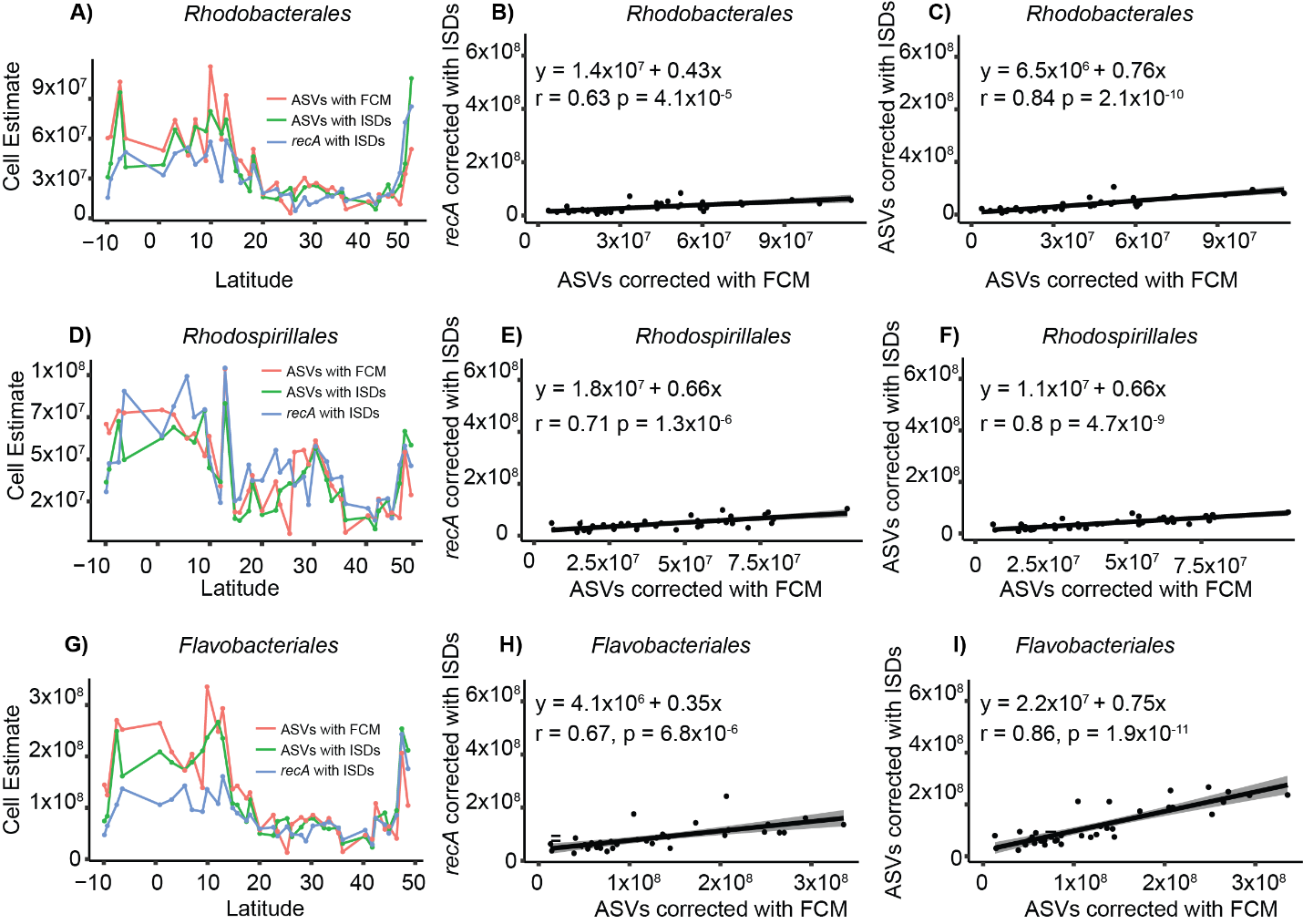
**

**Supplementary Figure 1. Copiotrophic bacteria (A, D, G)** Line plots of cell estimates per litre seawater of **(A)** *Rhodobacterales*, **(D)** R*hodospirialles*, **(G)** *Flavobacteriales* derived from three different methods. Red indicates estimates ASVs/L corrected with *Synechococcus* flow cytometry, green indicates ASVs/L corrected with internal standards and blue indicates metagenomically derived single copy *recA* genes (bacteria) per litre, corrected with internal standards. **(B, E, H)** are linear regressions of **(B)** *Rhodobacterales*, **(E)** R*hodospirialles*, **(H)** *Flavobacteriales* from metagenomically derived *recA*/*radA* cells/L corrected with internal standards compared to the same organism derived from ASVs/L corrected with flow cytometry. Finally **(C, F, I)** are linear regressions of **(C)** *Rhodobacterales*, **(F)** R*hodospirialles*, **(I)** *Flavobacteriales*, from ASVs/L corrected with internal standards compared to the same organism derived from ASVs/L corrected with flow cytometry.

*
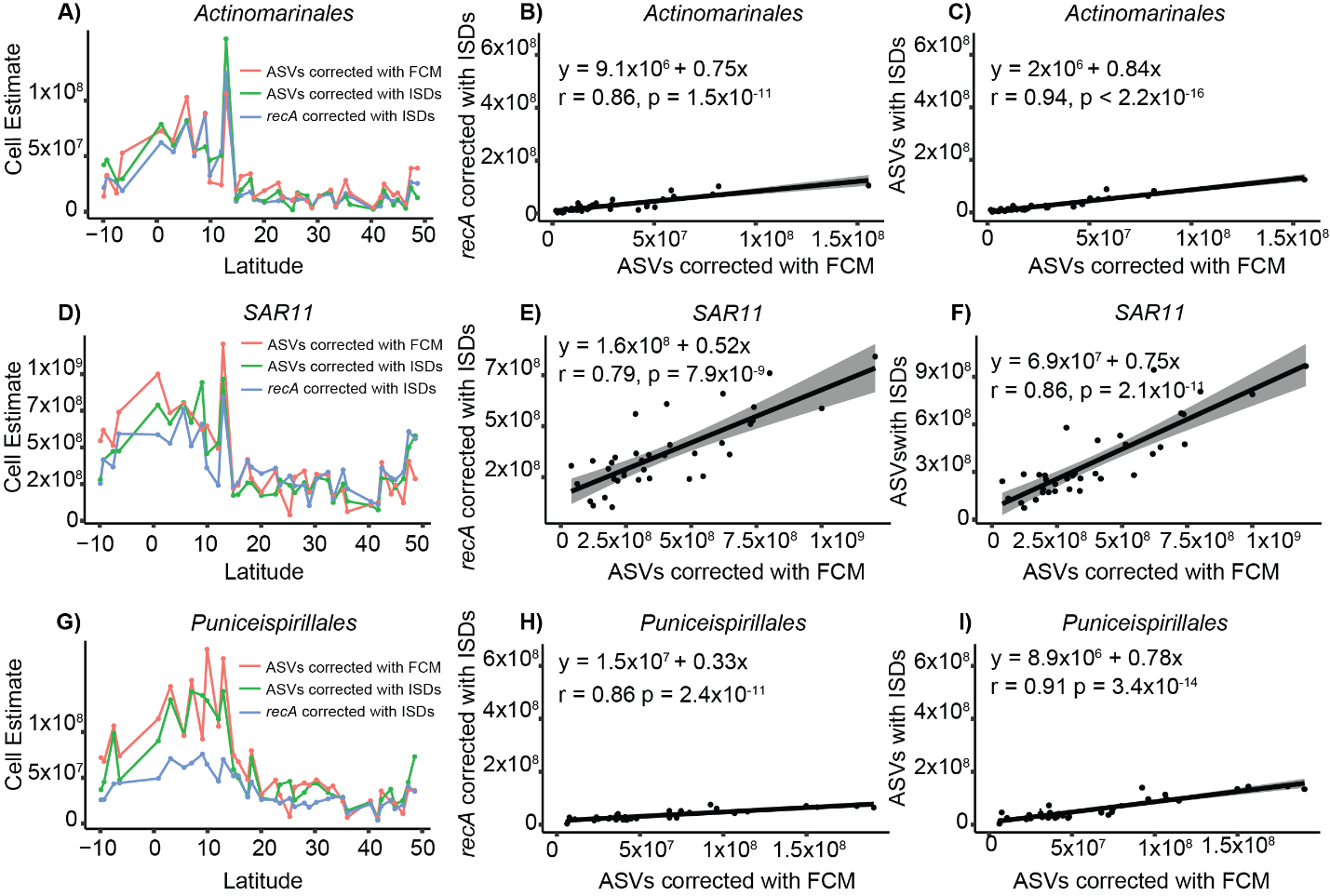
*

**Supplementary Figure 2. Oligotrophic bacteria (A, D, G)** Line plots of cell estimate per litre seawater of **(A)** *Actinomarinales*, **(D)** SAR11 and **(G)** *Puniceispiralles*, , derived from three different methods. Red indicates estimates ASVs/L corrected with *Synechococcus* flow cytometry, green indicates ASVs/L corrected with internal standards and blue indicates metagenomically derived single copy *recA* genes (bacteria) and single copy *radA* genes (archaea) per litre, corrected with internal standards. (**B, E, H)** are linear regressions of **(B)** *Actinomarinales*, **(E)** *SAR11* and **(H)** *Puniceispiralles*, from metagenomically derived *recA*/*radA* cells/L corrected with internal standards compared to the same organism derived from ASVs/L corrected with *Synechococcus* flow cytometry. Finally **(C, F, I, L)** are linear regressions of **(C)** *Actinomarinales*, **(F)** SAR11, and **(I)** *Puniceispiralles* from ASVs/L corrected with internal standards compared to the same organism derived from ASVs/L corrected with *Synechococcus* flow cytometry.


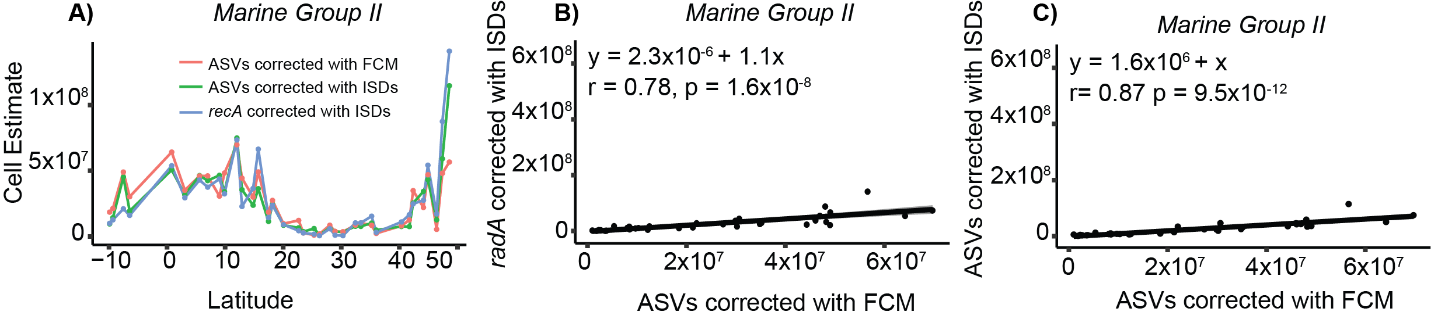


**Supplementary Figure 3. Archaea (A)** Line plots of cell estimate per litre seawater of MGII derived from three different methods. Red indicates estimates ASVs/L corrected with flow cytometry, green indicates ASVs/L corrected with internal standards and blue indicates metagenomically derived single copy *recA* genes (bacteria) and single copy *radA* genes (archaea) per litre, corrected with internal standards. (**B)** are linear regressions of MGII, from metagenomically derived *radA* cells/L corrected with internal standards compared to the same organism derived from ASVs/L corrected with flow cytometry. Finally **(C)** is a linear regression of MGII from ASVs/L corrected with internal standards compared to the ASVs/L corrected with flow cytometry.

**Supplementary Figure 4.** Line plots of ASV copies/L corrected with *Synechococcus* flow cytometry across latitude. **(A and B)** Show Bacteria, Archaea, and Eukaryotes from cruise **(A)** Gradients 2 and **(B)** Gradients 3. **(C and D)** Show *Actinomarinales*, *Flavobacteriales*, *Puniceispirillales*, *Rhodobacterales*, *Rhodospirillales*, *SAR11*, and *SAR86* from cruise **(C)** Gradients 2 and **(D)** Gradients 3. **(E and F)** Show *Prochlorococcus* and *Synechococcus* from cruise **(E)** Gradients 2 and **(F)** Gradients 3. **(G and H)** Show *Marine Group II Archaea* from cruise **(G)** Gradients 2 and **(H)** Gradients 3.

**
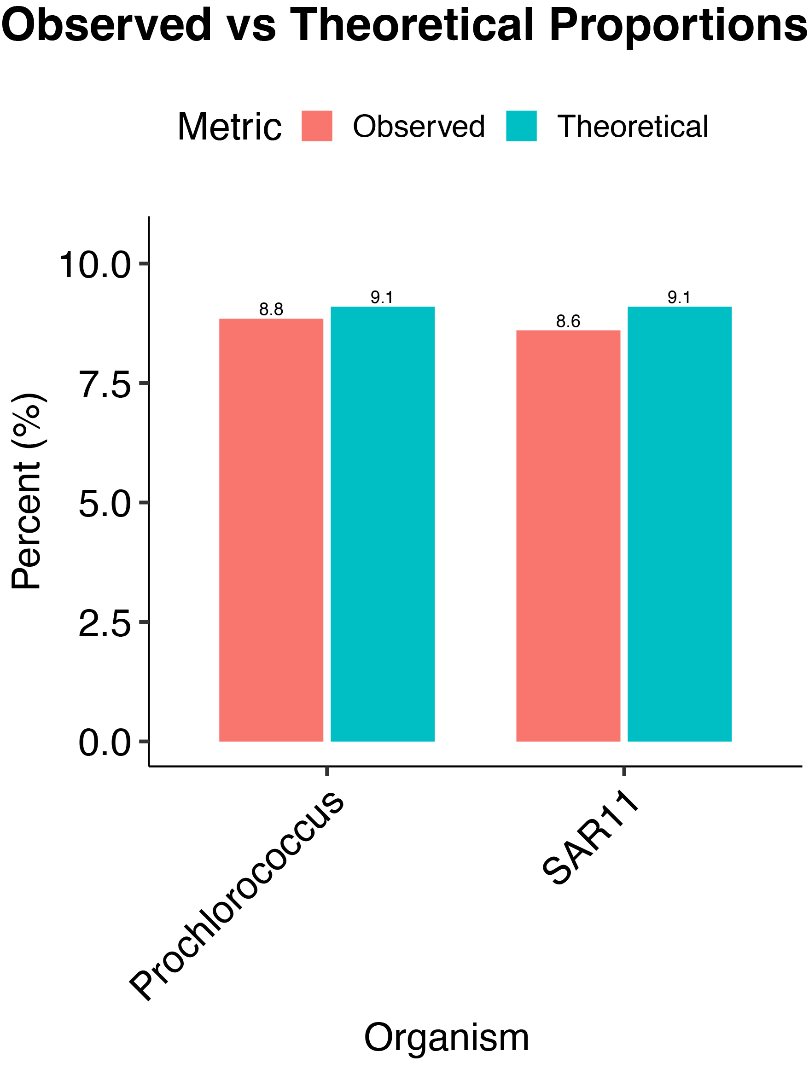
**

**Supplementary Figure 5.** Bar plot of observed verses expected proportions of *Prochlorococcus* and *SAR11* from mock communities.

Table 2. *Prochlorococcus* measured by flow cytometry, recA and amplicons by sample.

| SampleID | Prochlorococcus Flow Cytometry | Prochlorococcus recA | Prochlorococcus Amplicons (corrected w/ ISDs (copies/L) | Prochlorococcus Amplicons (corrected w/ FCM (copies/L) |
| --- | --- | --- | --- | --- |
| DNA-tag-02 | 8.80E+05 | 2.89E+06 | 1.37E+07 | 6.78E+06 |
| DNA-tag-03 | 4.37E+07 | 7.53E+07 | 6.13E+07 | 4.99E+07 |
| DNA-tag-04 | 8.94E+07 | 1.33E+08 | 8.24E+07 | 3.51E+07 |
| DNA-tag-05 | 6.30E+07 | 5.94E+07 | 3.82E+07 | 4.16E+07 |
| DNA-tag-06 | 8.68E+07 | 9.48E+07 | 8.31E+07 | 5.35E+07 |
| DNA-tag-07 | 9.17E+07 | 1.22E+08 | 7.92E+07 | 1.09E+08 |
| DNA-tag-08 | 9.78E+07 | 1.65E+07 | 6.63E+06 | 1.11E+07 |
| DNA-tag-09 | 1.54E+08 | 1.42E+08 | 9.31E+07 | 9.94E+07 |
| DNA-tag-10 | 1.71E+08 | 1.65E+08 | 1.19E+08 | 5.60E+07 |
| DNA-tag-11 | 1.74E+08 | 2.46E+08 | 1.68E+08 | 1.39E+08 |
| DNA-tag-12 | 1.82E+08 | 1.93E+08 | 1.42E+08 | 1.91E+08 |
| DNA-tag-13 | 1.53E+08 | 1.70E+08 | 1.36E+08 | 1.52E+08 |
| DNA-tag-14 | 6.37E+07 | 1.08E+08 | 1.12E+08 | 1.21E+08 |
| DNA-tag-15 | 9.45E+07 | 8.11E+07 | 1.43E+08 | 1.45E+08 |
| DNA-tag-16 | 1.39E+08 | 1.24E+08 | 1.49E+08 | 1.95E+08 |
| DNA-tag-17 | 6.68E+07 | 9.74E+07 | 7.18E+07 | 1.12E+08 |
| DNA-tag-18 | 3.76E+07 | 1.49E+08 | 1.72E+08 | 2.84E+07 |
| DNA-tag-19 | 1.13E+08 | 1.08E+08 | 1.48E+08 | 1.08E+08 |
| DNA-tag-20 | 1.53E+08 | 1.92E+08 | 1.14E+08 | 2.13E+08 |
| DNA-tag-21 | 2.86E+08 | 2.56E+08 | 1.73E+08 | 1.97E+08 |
| DNA-tag-22 | 3.55E+08 | 5.22E+08 | 5.21E+08 | 5.83E+08 |
| DNA-tag-23 | 5.06E+08 | 3.00E+08 | 2.19E+08 | 3.55E+08 |
| DNA-tag-24 | 3.01E+08 | 4.54E+08 | 3.21E+08 | 4.33E+08 |
| DNA-tag-25 | 3.07E+08 | 4.09E+08 | 4.07E+08 | 5.12E+08 |
| DNA-tag-26 | 5.05E+08 | 5.83E+08 | 6.60E+08 | 8.24E+08 |
| DNA-tag-27 | 1.69E+08 | 1.40E+08 | 2.56E+08 | 2.38E+08 |
| DNA-tag-28 | 4.28E+08 | 5.55E+08 | 5.67E+08 | 8.04E+08 |
| DNA-tag-29 | 2.39E+08 | 3.93E+08 | 5.18E+08 | 3.42E+08 |
| DNA-tag-30 | 2.41E+08 | 2.97E+08 | 4.18E+08 | 4.54E+08 |
| DNA-tag-31 | 2.29E+08 | 3.53E+08 | 4.11E+08 | 4.09E+08 |
| DNA-tag-32 | 3.94E+08 | 4.34E+08 | 5.03E+08 | 5.58E+08 |
| DNA-tag-34 | 3.80E+08 | 4.64E+08 | 5.82E+08 | 7.37E+08 |
| DNA-tag-37 | 2.87E+08 | 5.64E+08 | 5.28E+08 | 8.23E+08 |
| DNA-tag-38 | 2.50E+08 | 5.07E+08 | 7.23E+08 | 7.84E+08 |
| DNA-tag-39 | 3.34E+08 | 3.28E+08 | 3.57E+08 | 5.32E+08 |
| DNA-tag-40 | 3.25E+08 | 1.85E+08 | 2.03E+08 | 3.95E+08 |
